# Towards optoelectronic urea biosensors

**DOI:** 10.1007/s00216-014-8434-z

**Published:** 2015-01-27

**Authors:** Marta Pokrzywnicka, Robert Koncki, Łukasz Tymecki

**Affiliations:** Department of Chemistry, University of Warsaw, Pasteura 1, 02-093 Warsaw, Poland

**Keywords:** Biosensor, Bioreactor, Urease, Light-emitting diodes, Instrumentation, Flow analysis

## Abstract

Integration of immobilized enzymes with light-emitting diodes (LEDs) leads to the development of optoelectronic enzyme-based biosensors. In this work, urease, used as a model enzyme, immobilized in the form of an open-tubular microbioreactor or biosensing membrane that has been integrated with two red LEDs. It forms complete, fiberless, miniaturized, and extremely economic biooptoelectronic devices useful for nonstationary measurements under flow analysis conditions. Both enzyme-based biodevices, operating according to the paired emitter detector diode (PEDD) principle, allow relatively fast, highly sensitive, and well-reproducible urea detection in the millimolar range of concentrations. Potential analytical applications of the developed urea bioPEDDs have been announced. Both presented constructions will be easily adapted for the development of other optoelectronic biosensors exploring various enzyme-based schemes of biodetection.

**Electronic supplementary material:**

The online version of this article (doi:10.1007/s00216-014-8434-z) contains supplementary material, which is available to authorized users.

## Introduction

Since the 1980s, optical (bio)sensors were fabricated by integration of (bio)chemosensitive membranes with optical fibers in the form of dip probes (so-called FOCS) coupled with an external spectrometer. One of current trends in the development of optoelectronic devices for the needs of modern analytical chemistry is paired emitter detector diodes (PEDDs). According to the PEDD operation principle [1, 2], a complete optical detector consists of light-emitting diodes (LEDs) only. The simplest PEDD consists of two LEDs. One of them is applied in a conventional way as a source of light, whereas the second, operating in the reverse mode, plays the role of a light detector. In both cases, LEDs emit or detect light in quite narrow wavelength ranges, thus properly performed selection of LED emitter and LED detector leads to formation of a complete and selective optical detector. The use of monochromators, collimators, and optical fibers is eliminated. Obviously, such dedicated optoelectronic devices are extremely cheap and naturally predestined for miniaturization and integration with more sophisticated analytical systems. Moreover, selected LEDs are often integrated in the format of optical flow cells.

The first analytical PEDDs have been developed for photometric measurements as dedicated, economic, and miniaturized optoelectronic devices useful for conventional measurement with the use of cuvettes as well as for the use in flow analysis format (dedicated flow-through detectors for flow injection analysis (FIA), SIA, MCFA, and HPLC). Such photometric devices have been successfully applied for determination of selected metal ions [1–9], ammonia [10], inorganic anions [11–15], total organics [16], quinine [17], hemoglobin [18], creatinine [19], and proteins [20] as well as for enzyme activity assays [21–23]. Only recently, Nwankire et al. [24] have reported on PEDD-based microfluidic analysis system allowing blood assay of five analytes important for liver diagnostics (albumin, total and direct bilirubin, alkaline phosphatase, and γ-glutaral transferase).

Our research group has demonstrated that PEDDs can be also configured as complete and effective fluorimetric detectors consisting of integrated LED inductor and LED detector of fluorescence [17, 25]. Such approach is possible because LEDs are found to be partially selective detectors able to detect fluorescence emission with negligible effects from the exciting light. Until now, two and three LED-based fluorimetric detectors have been successfully developed for determination of calcium [25, 26] and phosphate [14, 15] ions, quinine [17], vitamin B [27], and proteins [20]. Only recently, turbidimetric [28–31] and nephelometric [30, 31] detectors operating according to PEDD principle have been reported. Such detectors have been applied for measurements of sample turbidity [28] as well as for detection of phosphates in drinks [29] and total protein level in physiological fluids (urine [30] and cerebrospinal fluid [31]).

A new intensively explored PEDD area is paired LEDs integrated with chemosensitive layers resulting in the development of a new class of optical chemical sensors. Recently, several photometric PEDD-based gas sensors based on immobilized pH indicators useful for detection of acidic vapors [32, 33], sweat [34], and carbon dioxide [35] have been developed. Another example is an optoelectronic flow-through redox sensor based on chemosensitive Prussian blue film, useful for determination of ascorbic acid and hydrogen peroxide [36]. Finally, also the first prototypes of fluorimetric PEDD-based sensors dedicated for the detection of riboflavin [27] and oxygen [37] have been demonstrated.

Until now, only one PEDD-based enzyme biosensor, developed for photometric detection of glucose, has been reported in the analytical literature [38]. In this short communication, we present how to integrate PEDDs with immobilized urease, used here as a model enzyme, to obtain optoelectronic urea biosensors. Two promising constructions of urea bioPEDDs based on urease bioreactor and urease-containing chemosensitive membrane will be demonstrated. Both biodevices are designed for measurements under FIA conditions.

## Experimental

### Reagents and materials

Urease isolated from Jack bean (EC 3.5.1.5, lyophilized powder 100 U/mg) and 1-ethyl-3-(3-dimethylaminopropyl)carbodiimide hydrochloride (EDAC) were obtained from Sigma (USA). High-molecular-weight carboxylated PVC was purchased from Aldrich (Germany). All other reagents, including bromothymol blue (BTB) and triacetate cellulose (TAC), solvents, and plasticizer of analytical grade, were obtained from POCh and used without further purification. Water used for experiments was distilled and passed through a Milli-Q purification system.

Red LEDs (*λ*_max_ = 630 nm, diameter = 5 mm; lens: transparent, flat front; view angle = 140°; average luminous intensity at 20 mA current supply = 1.5 Cd) were purchased from OptoSupply (Hong Kong; product symbol OSHR53E1A-LM). Polyether ether ketone (PEEK) was used as a construction material for LED and biocomponent arrangement. The body of devices was micromachined using manually operated milling machine and lathe.

### Urease-based bioreactor preparation

Bioreactors were prepared using 1/16-in. blue-coded nontransparent PVC tubing (Ark-Plast, product no. KH-95871-30) purchased from Cole-Palmer (USA). As proposed elsewhere [39], the tubing was coated with PVC–COOH by flowing through its solution in THF (60 mg/mL) and evaporation of solvent residues at room temperature. For enzyme immobilization, the reactor was filled with a water solution of urease (20 mg/mL) containing EDAC (10 mg/mL) and left at room temperature overnight. Before the first use, the bioreactor was washed for 2 h by passing through working buffer.

### Urease-based biosensing membrane preparation

pH-sensitive membranes were prepared according to a protocol given elsewhere [40]. Their composition was BTB (3 %), tridodecylmethylammonium chloride (TDMAC) (4 %), TAC (37 %), ethylene glycol (28 %), and dioctyl sebacate (DOS) (28 %). pH enzyme membranes were prepared in almost the same way, only before solvent evaporation from the added membrane cocktail solution urease (15 mg/mL). The final composition of obtained biomembrane was as follows: 2.4 % BTB, 3.3 % TDMAC, 23 % DOS, 23 % ethylene glycol, 30.5 % TAC, and 17.8 % of enzyme.

### Measurement setup

For supplying the LED emitter, the lab-made circuit was prepared with typical electronic components (TME, Poland). For recording of voltaic signal [41] generated by a LED detector, a multimeter from Axiomet (model AX-18B; China) connected with PC via a USB interface was applied. The optimal currents supplying LED emitter offering maximal sensitivity of measurements for bioPEDDs based on bioreactor and biomembrane were found to be 10.0 and 1.0 mA, respectively.

The simple double-channel FIA manifold applied for bioPEDD investigations, consisting of Gilson pomp (model Minipuls 3; France), Rheodyne injection valve (model 5020; USA), and PTFE Microbore Tubings (ID 0.8 mm) from Cole-Palmer (USA), is shown in the Electronic Supplementary Material (ESM) (Fig. S1). Water urea standards were injected into water line (injection volume 0.2 mL). The second line delivers 10 mM phosphate buffer, pH of 6.0. In case of investigations on bioreactor-based biosensor, the carrier buffer was additionally spiked with BTB (50 mg/L).

## Results and discussion

### Urea biodetection scheme

A large number of enzyme biosensing schemes are based on the detection of pH changes caused by proteolytic products of biocatalyzed reactions. Urease, a model enzyme in this research, exhibits maximal activity at pH of 6–8, whereas the products of biocatalyzed hydrolysis of urea cause alkalization of the reaction environment approximately up to pH of 9.3 [42]. pH changes in the range of 6–9 can be easily detected by measurement of BTB absorbance because p*K*_I_ of this indicator is 7.2. As shown in the Electronic Supplementary Material in this work, two ordinary red LEDs are sufficient for monitoring changes of BTB absorbance, because their emission and detection spectra are fully compatible with an absorption spectrum of blue alkaline form of this dye (ESM Fig. S2). The calibrations of the PEDD on BTB (ESM Fig. S3A) and on pH (ESM Fig. S3B) confirm that such measurements are highly sensitive (over 1000 mV of stationary signal). As reported elsewhere [43], a red-red PEDD-based cuvette photometer is useful for detection of several blue acid/base indicators of different p*K*_I_ values enabling wide range detection of pH. In this work, a model urease-pH-BTB biosensing scheme for optical urea detection will be applied for flow-through bioPEDD development.

### BioPEDD based on bioreactor

**Fig. 1:**
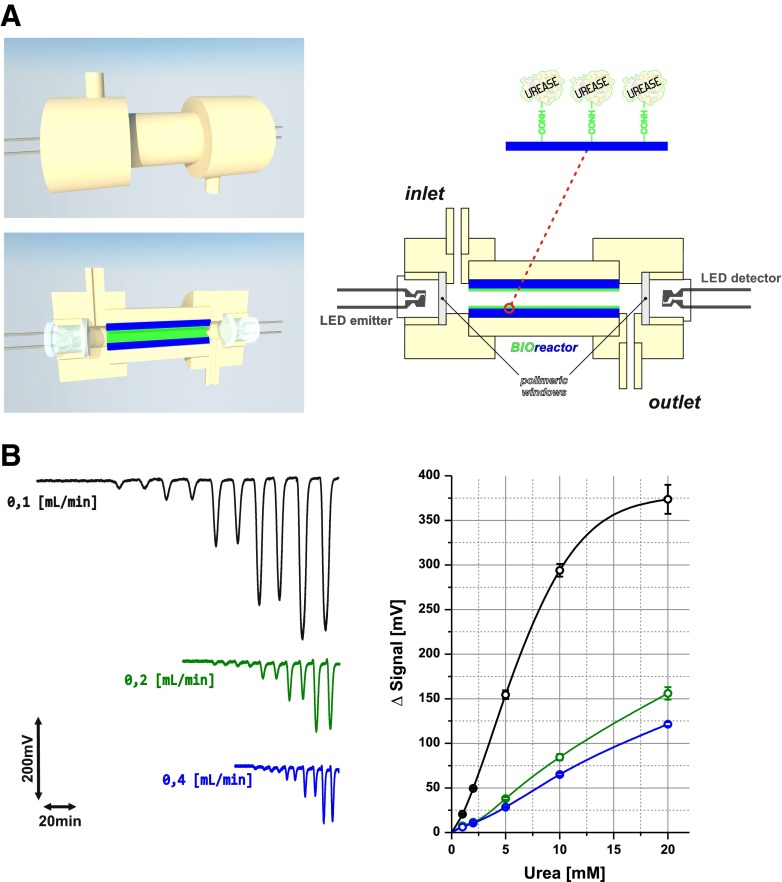
Design of bioPEDD based on bioreactor (**A**) and calibration FIA grams obtained at different flow rates (given in the figure) with corresponding calibration graphs (**B**)

PEDD integrated with a bioreactor is depicted in Fig. 1A. For this study, a plastic open-tubular reactor with chemically immobilized urease has been applied. According to immobilization protocol reported elsewhere [39], the inner walls of PVC tubing have been coated with carboxylated PVC and the functional groups have been applied for covalent binding of urease molecules using a one-step carbodiimide method. The dimensions of bioreactor (90 μL of inner volume of 2.0-cm-long tubing) mounted between LEDs define both the internal volume of the resulting flow cell and the optical path length for photometric measurement. The construction shown in Fig. 1A allows easy replacement of bioreactor. In the course of 1-year-long experimental work, the exchange of LEDs was not necessary. The paired LEDs measure (directly inside the bioreactor) changes of absorbance of BTB, permanently present in the flowing carrier buffer. These changes are proportional to the alkalization of carrier by the products of urea hydrolysis. Figure 1B presents recordings of biosensor calibration performed at different flow rates and corresponding calibration graphs. The baseline generated by the system is stable over time, and the peaks are well reproducible. An increase of flow rate causes both a decrease of sensitivity and an increase of sample throughput. In all cases, the bioPEDD is useful for urea determination in the millimolar concentration range.

### BioPEDD based on biosensing membrane

**Fig. 2:**
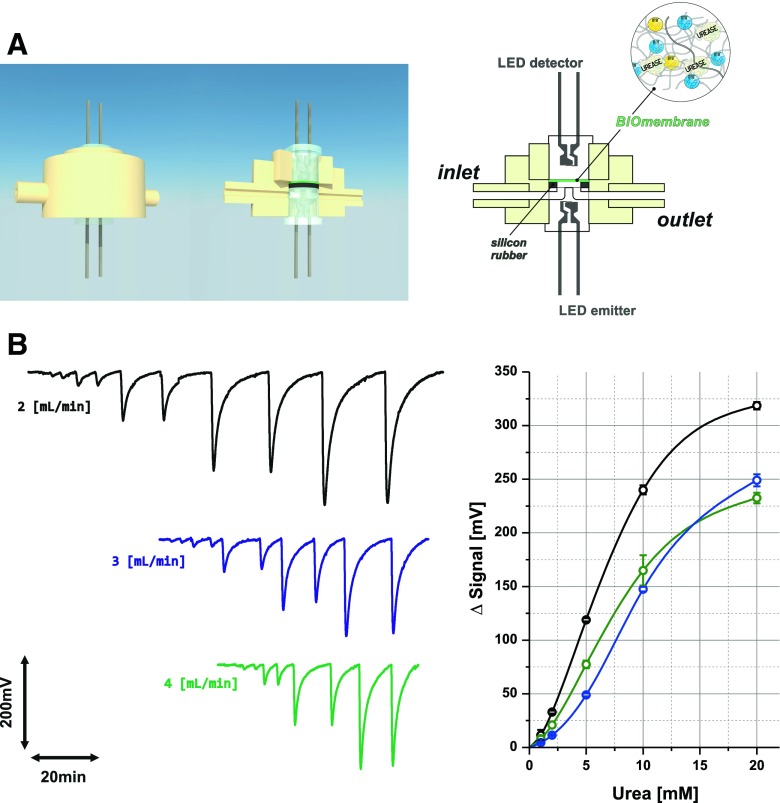
Design of bioPEDD based on biosensing membrane (**A**) and calibration FIA grams obtained at different flow rates (given in the figure) with corresponding calibration graphs (**B**)

The second kind of bioPEDD contains optically pH-sensitive membrane with BTB immobilized in plasticized TAC matrix [40]. The sensitivity and stability of such PEDD-based flow-through pH sensor is illustrated in Electronic Supplementary Material Fig. S4. As reported elsewhere [44], TAC membranes deposited by evaporation of solvent are an effective matrix for physical immobilization of urease by enzyme inclusion. The design of the developed flow-through PEDD is shown in Fig. 2A. In this bioPEDD, the replacement of biosensing membrane also is simple and the exchange of LEDs is not necessary. Calibrations of the resulting urea biosensor performed at different flow rates are shown in Fig. 2B. The baseline generated by the system is stable over time, and the peaks are well reproducible. The shape of recorded peaks clearly evidences the memory effect of sensing membrane. An increase of flow rate causes both a decrease of sensitivity and an increase of sample throughput; however, this effect is not as strong as in the case of bioreactor-based bioPEDD (see Fig. 1B). As previously, the biosensor allows urea determination in the millimolar range of concentrations.

### Analytical performance of developed urea bioPEDDs

The quantitative analytical parameters of both developed bioPEDDs are collected in Table 1. The offered ranges and limits (limit of detection and limit of quantification values were determined as a standard deviation of the blank signals multiplied by 3 or 10, respectively) for urea determination are similar and comparable with those reported in the literature for conventional optical [45, 46] and potentiometric [44, 47, 48] pH-based urea biosensors. The analytical characteristics like sigmoidal shape of calibration graphs as well as effects from flow rate and concentration and pH of buffer are consistent with theoretical predictions based on the model of pH enzyme-based biosensors [42].

**Table 1:** Analytical parameters of the developed urea bioPEDDs

Flow rate (mL/min)	Dynamic range (DR) (mM)	Sensitivity in DR (mV/mM)	Linear range (LR) (mM)	Sensitivity in LR (mV/mM)	*R* ^2^	Limit of detection (mM)	RSD % (for 5 mM urea)	Baseline drift (mV/h)	Injection frequency (sample/h)
Bioreactor-based bioPEDD									
0.1	0.6–25	15.2	2–10	30.2	0.990	0.35	2.3	1.3	4
0.2	1.8–60	5.3	2–20	8.4	0.989	0.86	1.8	3.0	6
0.4	2.1–80	4.5	1–30	6.6	0.998	0.90	2.4	0.7	10
Biosensing membrane-based bioPEDD									
2.0	0.9–35	10.0	1–10	25.1	0.998	0.69	1.8	2.9	3
3.0	1.4–50	6.5	2–10	17.0	0.992	0.97	5.6	3.4	4
4.0	2.9–50	6.5	5–10	19.7	0.999	2.66	3.0	4.7	5–6

Significant difference between developed bioPEDDs is in operational stability. For bioreactor-based biodevice after 7 days of continuous flow measurements, over 60 % of initial sensitivity was retained. The analytical properties of bioPEDD stored dry under ambient conditions within 6 months (storage stability test) did not change. Due to the applied dye and enzyme immobilization method, the lifetime of biomembrane-based bioPEDD is significantly shorter. After 3 days of continuous flow measurements (operational stability test), only 50 % of initial sensitivity was observed. Moreover, similarly as in case of pH sensor (see Electronic Supplementary Material Fig. S4), the baseline drift caused by BTB leaching is observed. On the other hand, both pH membranes and pH enzyme membranes after a half year of dry storage under ambient conditions retain full sensitivity and all mechanical properties. It is obvious that biosensing membranes with covalently bound enzymes [45, 46], additionally based on highly lipophilic [45] or insoluble [46] pH-indicative dyes, will exhibit significantly better operational stability.

It is worth to notice that the hybrid construction of bioPEDD obtained by integration of bioreactor, pH-sensitive membrane, and LED system in the form of flow-through cell was also tested; however, the obtained analytical results were not satisfactory. Such biosensor exhibited long response time limited by the dynamics of pH membrane (Electronic Supplementary Material Fig. S1) and significantly lower sensitivity caused by changes of pH in the bulk solution. These changes are smaller than those obtained when the enzyme reaction process takes place directly inside a pH-sensitive membrane.

## Conclusions

The main goal of this short communication is the presentation of two concepts of the integration of optoelectronics with immobilized enzymes leading to the development of smart, miniaturized, and highly economic biosensors designed for practical flow analysis. In our opinion, the demonstrated constructions are easily adaptable for many other enzyme-based biosensing schemes coupled with optical detection as well as for several kinds of open-tubular bioreactors and biosensing membranes developed for biorecognition of various analytes.

The results shown in this note have a demonstrative character, without focusing on real analytical applications. The operation principles of pH-based enzyme biosensors [42] limit the practical use of biodevices presented in this work to analysis of samples having relatively low and constant buffer capacity like urine and saline extracts from pharmaceutical ointments [48]. However, it was reported that urea biosensors operating according to the applied detection scheme are also useful for analysis of postdialysate fluids [46, 47] produced by an artificial kidney in the course of hemodialysis (HD). The significance of monitoring HD therapy and urea kinetic modeling of HD treatments has been discussed in details elsewhere [49]. The study on the application of urea bioPEDDs reported in this note for such biomedical needs is continued in the clinical settings.

## Electronic supplementary material

Below is the link to the electronic supplementary material.

ESM 1 (PDF 333 kb)
