# Supplementary material for: Towards optoelectronic urea biosensors
Source: Anal Bioanal Chem. 2015 Jan 27;407(7):1807–12. doi: 10.1007/s00216-014-8434-z (PMC4336418; doi:10.1007/s00216-014-8434-z)
Supplement: Supplementary file 1 — (PDF 333 kb) [file 216_2014_8434_MOESM1_ESM.pdf]

Analytical and Bioanalytical Chemistry

Electronic Supplementary Material

Towards optoelectronic urea biosensors

by Pokrzywnicka M,. Koncki R and Tymecki Ł

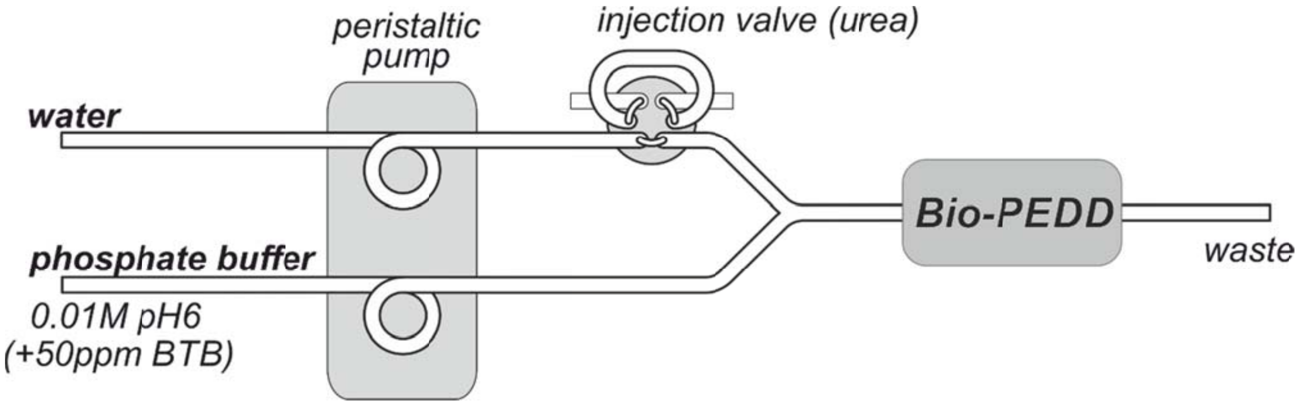

Figure S1. Scheme of FIA setup for testing of developed urea bioPEDDs.

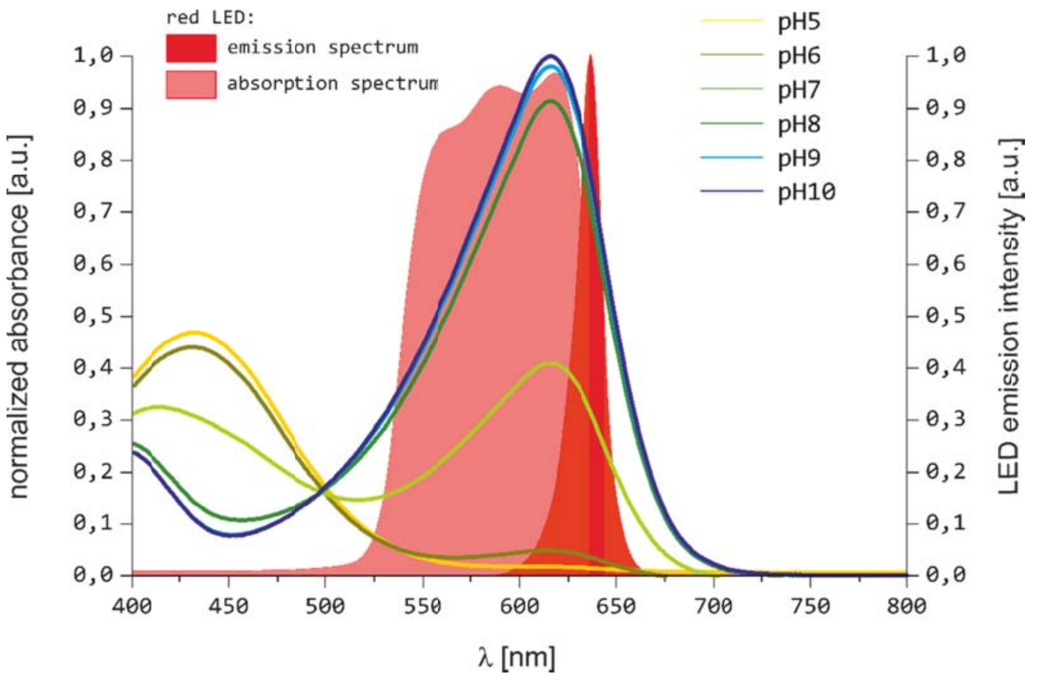

Figure S2. BTB spectra recorded at different pHs and emission and detection spectra for red LED

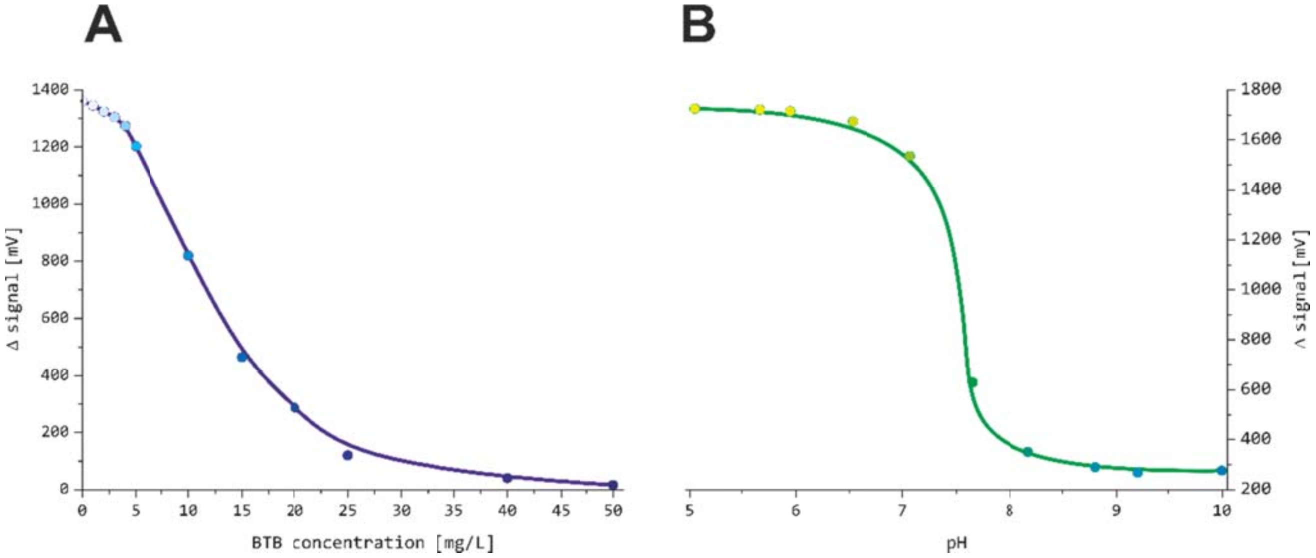

Figure S3. PEDD calibration on BTB (A) and pH (B).

The PEDD measurements were performed using conventional 1-cm plastic cuvettes (Sarstedt 67.740, Germany) in the cuvette holder with mounted LEDs at the optimal current supplying LED-emitter (25 mA), offering maximal sensitivity of measurements.

Fig. S2 - the spectra were recorded using Optic Ocean spectrometer, model USB 2000

Fig. S3A - BTB standards prepared in 0.01M borate buffer, pH of 9.2, Equation of the calibration curve in linear range (4-15mg/L) is as follow: y= -56.6x + 1440 (R square coefficient = 0.963)

Fig. S3B - 50 ppm BTB solutions in the series of universal buffers have been used.

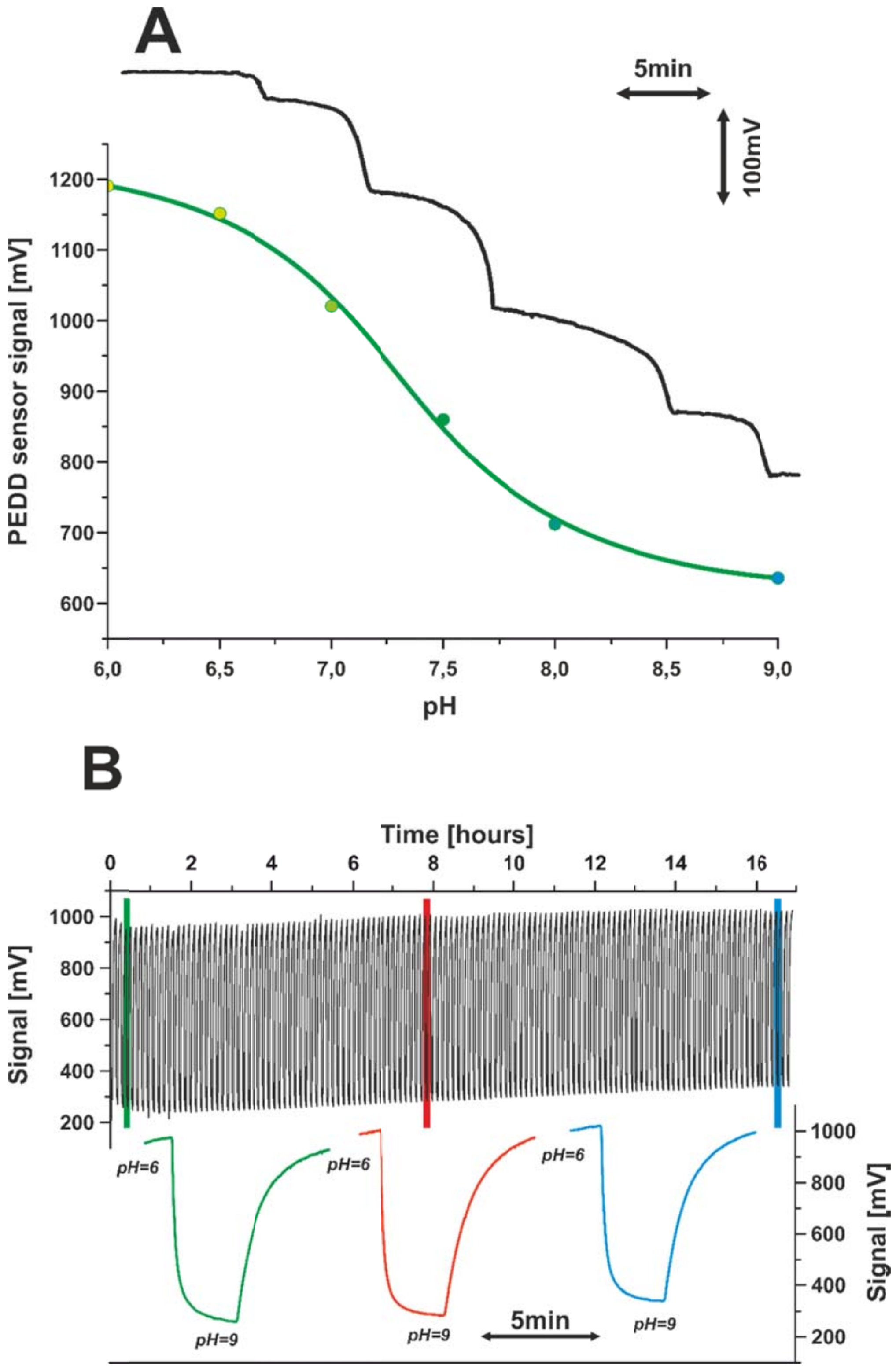

Figure S4. Calibration graph for pH-sensitive PEDD obtained under continuous flow conditions with corresponding calibration graph (A) and its long-term operational stability test under FIA conditions (B).

Fig. S4A - measurements performed using phosphate buffers.

Fig. S4B – in case of operational stability test 0.01M phosphate buffer (pH 6) and 0.010M borate buffer (pH 9) were pumped by turn as a carrier.
